# A novel cyclic pentapeptide, H-10, inhibits B16 cancer cell growth and induces cell apoptosis

**DOI:** 10.3892/ol.2014.2121

**Published:** 2014-05-08

**Authors:** GENG ZHANG, SHOUXIN LIU, YUNJIANG LIU, FEIFEI WANG, JINGWEN REN, JIFENG GU, KAIXUAN ZHOU, BAOEN SHAN

**Affiliations:** 1 Research Center, The Fourth Hospital of Hebei Medical University, Shijiazhuang, Hebei 050011, P.R. China; 2 State Key Laboratory Breeding Base-Hebei Province Key Laboratory of Molecular Chemistry for Drug, Hebei University of Science and Technology, Shijiazhuang, Hebei 050018, P.R. China; 3 Breast Center, The Fourth Hospital of Hebei Medical University, Shijiazhuang, Hebei 050011, P.R. China

**Keywords:** sansalvamide A, B16, apoptosis, caspase-3

## Abstract

Sansalvamide A, a cyclic depsipeptide isolated from a marine fungus of the *Fusarium* genus, exhibits significant antitumor activity. In the present study, H-10 (molecular formula, C_38_H_55_N_5_O_6_; molecular weight, 677.8732), a novel sansalvamide A derivative, demonstrated an inhibitory effect on the proliferation of murine melanoma B16 cells. It was confirmed that H-10 induced the apoptosis of the B16 cells. The inhibitory rate of various concentrations of H-10 on the B16 cells was measured by sulforhodamine B colorimetric assay, and the results revealed that the inhibitory effect of H-10 on the B16 cells occurred in a concentration-dependent manner. In addition, a growth curve model of the B16 cells treated with 50 μM H-10 revealed that the effect of H-10 also occurred in a time-dependent manner. The apoptotic morphology of the B16 cells was observed using an optical microscope. Following the treatment of the cells with 50 μM H-10 for 24 h, the cell apoptosis rate was analyzed using flow cytometry. The expression levels of caspase-3, -8 and -9 were analyzed by western blot analysis, and the results indicated that H-10 may induce the apoptosis of B16 cells.

## Introduction

**Figure 1:**
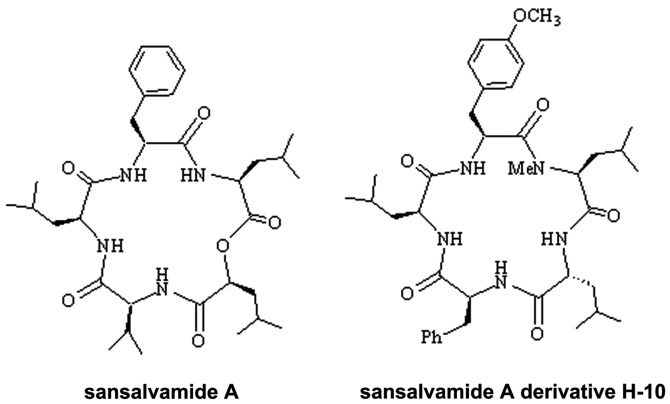
Structure of sansalvamide A and its derivative, H-10.

Malignant melanoma is a cancer with an increasing incidence and mortality rate. Furthermore, it is not sensitive to radiotherapy or chemotherapy, and thus presents a problem with regard to clinical treatment. Therefore, a number of studies have focused on the development of efficient and sensitive anticancer drugs. Sansalvamide A, a cyclic depsipeptide derived from a marine fungus (*Fusarium)*, has been found to exhibit significant antiproliferative effects in the National Cancer Institute’s 60 cancer cell line panel (1,2). In recent years, the synthesis of sansalvamide A derivatives have received increasing attention. Novel sansalvamide A derivatives show improved anticancer abilities (3), suggesting that these novel compounds may prove to be valuable therapeutic agents. In the present study, a novel sansalvamide A derivative, H-10, a cyclic pentapeptide (molecular formula, C_38_H_55_N_5_O_6_; molecular weight, 677.8732; Fig. 1), was investigated. Furthermore, this study focused on the effects of H-10 on the growth and apotosis of rat malignant melanoma B16 cells. The results may provide a basis for future sutides of this novel compound.

## Materials and methods

### Materials

RPMI 1640, trypsin-EDTA solution and fetal bovine serum (FBS) were purchased from Gibco-BRL (Carlsbad, CA, USA). H-10 cells were provided by the Hebei Province Key Laboratory of Molecular Chemistry for Drug (Shijiazhuang, China). Sulforhodamine B (SRB) was purchased from Tokyo Chemical Industry Co., Ltd (Tokoyo, Japan), and the bicinchoninic acid (BCA) kit was purchased from Shanghai Generay Biotechnology Co., Ltd. (Shanghai, China). The polyvinylidene fluoride (PVDF) membranes were purchased from Shanghai Generay Biotech Co., Ltd. The antibody against GAPDH (polyclonal rabbit anti-mouse) was purchased from Hangzhou Goodhere Biotechnology Co., Ltd. (Hangzhou, China). The antibodies against caspase-8, -9 and -3 (all polyclonal rabbit anti-mouse) were purchased from Bioworld Technology, Inc. (Minneapolis, MN, USA). The secondary fluorescence antibody (polyclonal goat anti-rabbit) was purchased from Nanjing Gene Biotech Co., Ltd. (Nanjing, China) The B16 cell line was stored at the Research Center of the Fourth Hospital of Hebei Medical University (Shijiazhuang, China).

### Cell culture

The cells were cultured in RPMI 1640 medium with 10% heat-inactivated FBS and 100 μg/ml penicillin and streptomycin. The cell line was grown in 25-cm^2^ flasks in a humidified atmosphere of 5% CO_2_ at 37°C, and the media were changed every second or third day. At 80–90% confluence, the cells were digested with trypsin-EDTA and plated in 25-cm^2^ flasks with media changes every second or third day, on 24- or 96-well plates.

### Concentration-dependent effect of H-10 on B16 cell growth inhibition

H-10 was dissolved in dimethyl sulfoxide (DMSO) and diluted with serum-free medium to prepare solutions of 1,000, 500, 100, 10 and 1 μM. Single cell suspensions of B16 cells were prepared and adjusted to the indicated concentration. The cells were then inoculated in 96-well plates (90 μl per well), with ~2,000 cells/well. Following 4 h of cell adherence, 10 μl H-10 was added to each well to form final concentrations of 100, 50, 10, 1 and 0.1 μM. Each group was placed into three wells, while a 1% DMSO group was simultaneously prepared as the control. The SRB colorimetric method was used to calculate the percentage growth of the B16 cells treated with the various concentrations of H-10 for 48 h.

### SRB colorimetric method

Following treatment with H-10 for 48 h, the cells were fixed with trichloroacetic acid (TCA) and the intracellular protein was stained with SRB. A total of 100 μl Tris base was then added to each well, and the dissolved SRB was detected using a microplate reader (Thermo Fisher Scientific, Vienna, Austria), whereby the values indirectly reflected the numbers of living cells. The medium in the 96-well plates was discarded and 100 μl TCA was added at a temperature of 4°C for 30 min. Next, the TCA was discarded and the cells were washed three times in distilled water, prior to being dried at room temperature for 1 h. A total of 100 μl 0.4% SRB was then added and the cells were agitated for 20 min. Next, the dye solution was discarded, and the cells were washed three times with 1% acetic acid and dried at room temperature for >6 h. Finally, 100 μl Tris base was added and cells were agitated for 5 min. The optical density was recorded at a wavelength of 490 nm using a microplate reader.

### Time-dependent effect of H-10 on B16 cell growth inhibition

At 80–90% confluence, the cells were harvested with trypsin, and serum-free medium was used to produce a single-cell suspension. The cells were then seeded in 24-well plates at the concentration of 20,000 cells/well. After 24 h, the wells were replaced with fresh medium, including FBS. Next, the wells were treated with 50 μM H-10 and the cell numbers were counted following 24, 48, 72, 96, 120 and 144 h. A control group was prepared simultaneously and a growth curve was generated.

### Flow cytometric analysis of apoptotic cell death

At 80–90% confluence, the cells were treated with 50 μM H-10 for 24 h, while a control group was prepared. The treated and untreated cells were then harvested, washed with phosphate-buffered saline (PBS) and fixed with 70% ethanol for 24 h. Next, the cells were centrifuged at 300 × g for 5 min and the pellet was resuspended in PBS containing 50 μg/ml propidium iodide and 10 μg/ml RNase A. The cells with <2N DNA content were classified as apoptotic cells.

### Detection of caspase-8, -9 and -3 expression by western blot analysis

The cells at 80–90% confluence were treated with 10, 30 and 50 μM H-10 for 24 h, and a control group was prepared. The cellular protein was extracted by radioimmunoprecipitation assay lysis buffer and the concentration was measured using the BCA kit. A total of 50 μg protein was electrophoretically separated on a 10% polyacrylamide gel. The proteins were then transferred to a PVDF membrane under the conditions of 90 V and 200 mA for 60 min. Next, the membranes were incubated in antibody dilution solution (rabbit anti-mouse caspase-3, -8, -9 and GAPDH; 1:500) overnight at 4°C. The blots were then incubated with the secondary fluorescence antibody (1:5,000) for 2 h in the dark, and the results were detected using the Odyssey infrared imager (LI-COR, Inc., Lincoln, NE, USA).

### Statistical analysis

Statistical analysis was performed using SPSS version 13.0 software (SPSS, Inc., Chicago, IL, USA). Data are presented as the mean ± standard error of the mean and were analyzed by t-test. P<0.05 was considered to indicate a statistically significant difference.

## Results

### H-10 exhibits a concentration-dependent effect on B16 cell growth

**Figure 2:**
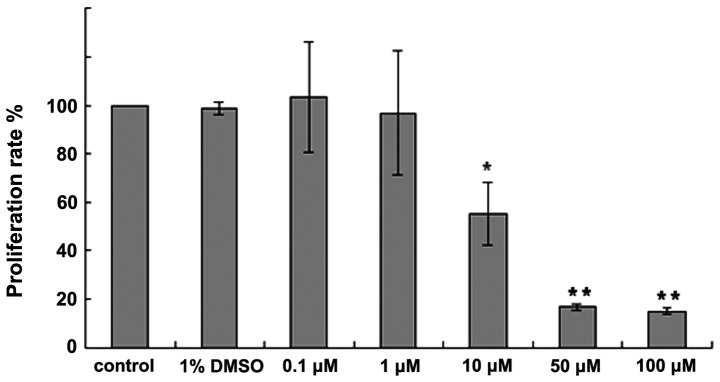
Effect of H-10 on the proliferation of B16 cells measured using the sulforhodamine B colorimetric method. Compared with the control group, no significant difference was identified in the proliferation rate of the 1% DMSO group (P>0.05). H-10 was found to cause concentration-dependent inhibition of the B16 cells. ^**^P<0.01; ^*^P<0.05 vs. control.

**Figure 3:**
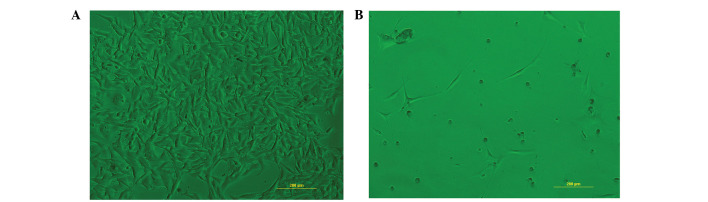
(A) B16 cells of the control group exhibiting vigorous and homogenous growth. Magnification, ×100. (B) B16 cells treated with 50 μM H-10 for 48 h exhibiting marked morphological changes, including decreased cell density, separation of the adjacent cells, rounding of the cells and cell shrinkage. Magnification, ×100.

Compared with the control group, no significant difference was identified in the proliferation rate of the 1% DMSO group (P>0.05). Following the treatment of the B16 cells with the various concentrations of H-10 (0.1, 1, 10, 50 and 100 μM) for 48 h, the proliferation rate of the B16 cells was found to gradually decrease. Furthermore, compared with control group, the proliferation rate of the B16 cells treated with 100, 50 and 10 μM H-10 was found to significantly decrease (P<0.01 for 100 μM; P<0.01 for 50 μM; and P<0.05 for 10 μM; Fig. 2). The morphological changes were observed under light microscopy (Fig. 3). B16 cells treated with 50 μM H-10 for 48 h exhibited marked morphological changes, including decreased cell density, separation of the adjacent cells, rounding of the cells and cell shrinkage.

### H-10 exhibits a time-dependent effect on B16 cell growth

**Figure 4:**
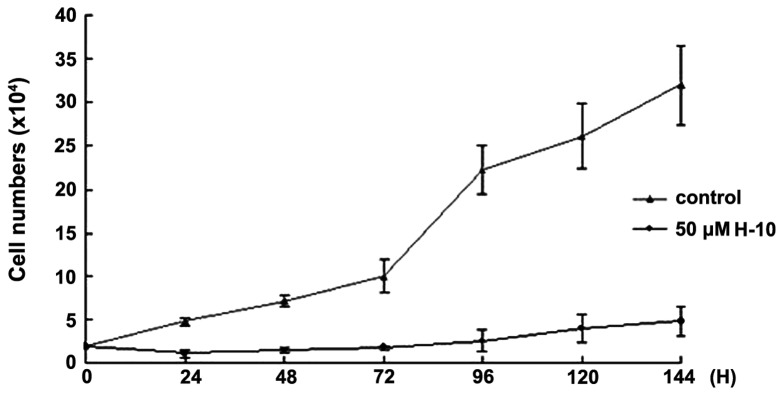
Time-dependent effect of 50 μM H-10 on B16 cell growth. The upper line of the graph presents the control group without H-10 treatment and the bottom line presents the group treated with H-10. At the end of each time-period, the cells were trypsinized to produce a single cell suspension and the cell number was counted. Data are presented as the mean ± standard error of the mean.

The time-dependent effect of H-10 on cell proliferation was measured by cell number. Following treatment with 50 μM H-10 for 24, 48, 72, 96, 120 and 144 h, the B16 cell numbers were counted and compared with the control group. The results indicated that H-10 inhibited the growth of the B16 cell line in a time-dependent manner (Fig. 4).

### H-10 induces the apoptosis of B16 cells

**Figure 5:**
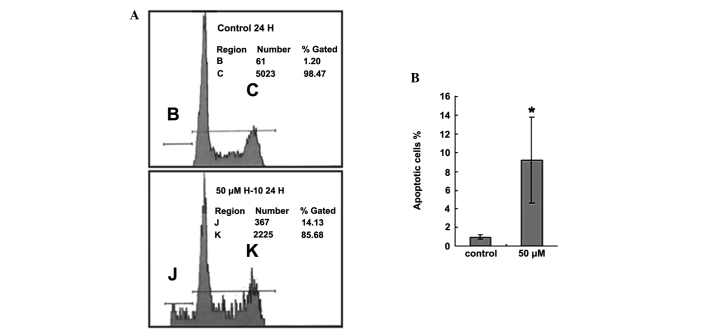
(A) Representative flow cytometry results showing the effect of H-10 on inducing B16 cells apoptosis. (B) Bar graph showing the percentage of apoptotic cells in the control and H-10-treated groups. Data are presented as the mean ± standard error of the mean. ^*^P<0.05 vs. control group.

Flow cytometry analysis of the cell samples demonstrated the ability of H-10 to induce apoptosis. Apoptotic cells were defined as those with subdiploid DNA content and were presented as the percentage of all counted cells per sample. The proportion of apoptotic cells in the untreated B16 cell group was 0.96±0.22%. However, in the group treated with 50 μM H-10 for 24 h, the percentage of apoptotic cells was 9.21±4.62% and this difference was found to be statistically significant (P<0.05; Fig. 5).

**Figure 6:**
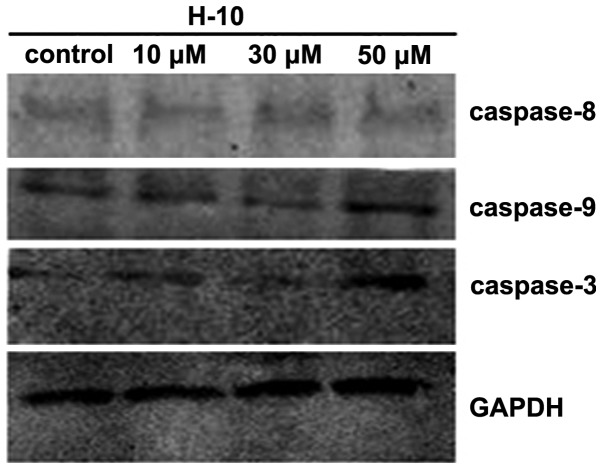
Western blot analysis of caspase-3, -8 and -9 expression. The results revealed an ascendant trend in the expression of caspase-3 and -9. However, no significant difference in caspase-8 expression was identified.

Caspase-3 plays a key role in cell apoptosis and is significant in its initiation. In the present study, the expression of caspase-3 was analyzed by western blot analysis, which further confirmed that H-10 induced apoptosis. Following treatment with 10, 30 and 50 μM H-10, the expression of caspase-3 in the B16 cells was found to increase with the concentration (Fig. 6).

### H-10 induces apoptosis of B16 cells via a mitochondrial pathway

The results of the western blot analysis revealed an ascendant trend in the expression of caspase-3 and -9. However, no significant difference was identified in the expression of caspase-8 among the control and test groups (Fig. 6). These results indicated that H-10 induces the apoptosis of B16 cells via a mitochondrial pathway.

## Discussion

Malignant melanoma is a cancer with an increasing incidence, high malignancy and poor prognosis. This cancer is characterized by its strong resistance and high metastasis and mortality rates. At present, no effective methods or drugs have been identified for treatment, and thus, novel methods are desperately required. Sansalvamide A has been found to exhibit marked antitumor effects by the National Cancer Institute’s 60 cancer cell line panel (1). Following numerous years of study, a variety of sansalvamide A derivatives have been synthesized and demonstrated to exhibit evident antitumor activity and improved stability. Compared with the linear peptide, sansalvamide and its derivatives cyclic structures may resist attack by exopeptidases and exhibit increased stability (4–6). Furthermore, sansalvamide A and its analogues are lipophilic, and thus exhibit rapid membrane absorption (7). Due to a specific cyclic peptide structure, the bioactive mechanism of sansalvamide A and its derivatives is extremely complicated. Under the action of different enzymes, the products of cyclic peptides are more complex than linear peptides. However, the amino acid sequences are different, and thus, the cyclic peptide is converted into various linear peptides, which may perform different functions. Compared with normal cells, malignant tumor cells exhibit increased reproductive activity and more complex enzyme and ligand-receptor signal transduction systems to maintain their specific biological behavior, which present as potential targets for sansalvamide A and its derivatives (8). Further study is required to explain the effects of these compounds on the specific signaling pathways.

Malignant melanoma cells exhibit enhanced survival and proliferation capabilities. One of the most important reasons for this is their antiapoptosis ability, which is the predominant problem for clinical chemotherapy drug tolerance. Therefore, the identification of an effective drug has become the focus of melanoma treatment. The results of the present study revealed that the proliferation rate of B16 cells is significantly inhibited by various concentrations of H-10 (0.1, 1, 10, 50 and 100 μM). Following the treatment of the B16 cells with 50 μM H-10 for 48 h, the cell proliferation rate was only 16.7%. In addition, the time-dependent test confirmed that H-10 exhibited a long-lasting suppressive effect on the B16 cell line.

Caspase-3 is the key enzyme in the execution of apoptosis, playing a significant role in the process. The initiation of cell apoptosis predominantly occurs via two routes, the exogenous death receptor and endogenous mitochondrial pathways (9,10). The exogenous pathway is initiated by death receptors, which then activate caspase-8. The endogenous pathway is initiated by ultraviolet radiation, the disappearance of growth factors or trophic factors, or various other stimulation factors. These lead to the release of cytochrome *c* into the cytoplasm from the mitochondria, and the subsequent activation of caspase-9. Procaspase-3 is subsequently hydrolyzed and activated by caspase-8 or -9. In the present study, the expression of caspase-3, -8 and -9 was detected following treatment with the various concentrations of H-10 (10, 30 and 50 μM), and the results of western blot analysis revealed an ascendant trend in the expression of caspase-3 and -9. However, no significant difference was identified in caspase-8 expression among the control and test groups. These results support the hypothesis that H-10 may inhibit the growth of B16 cells via mitochondrial pathway-induced apoptosis.

In conclusion, the results of the present study indicate that H-10 may induce the apoptosis of B16 cells. Considering the chemoresistance exhibited by melanoma towards conventional chemotherapy drugs, this novel compound may provide promising improvements in the therapeutic approach to melanoma treatment.
